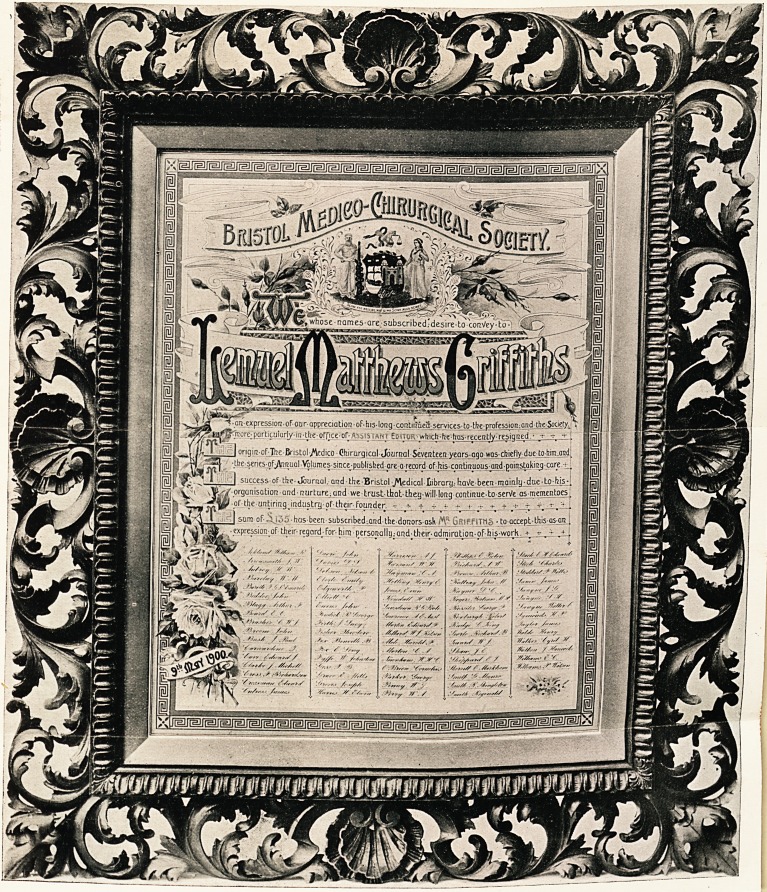# Presentation to Mr. L. M. Griffiths

**Published:** 1900-06

**Authors:** 


BRISTOL MEDICO-CHIRURGICAL SOCIETY.

### PRESENTATION TO MR. L. M. GRIFFITHS.

The meeting of the Bristol Medico-Chirurgical Society of May 9th, 1900, was a memorable occasion in its history.

The President, Mr. W. H. Harsant, said that a meeting of the Committee of the Society was called to consider the most suitable means of expressing their sense of indebtedness to Mr. L. M. Griffiths on his retiring from the post of Assistant-Editor of the *Journal*, and he would call upon Dr. Shingleton Smith to make a statement.

Dr. Shingleton Smith felt his duty was a very pleasurable one. On January 13th a meeting of the Committee took place, when he was requested to act as Treasurer, and he had now the pleasure to report that their suggestions had met with a most gratifying and liberal response. Various contributions had been received from eighty-two subscribers, amounting to £135 16s. It was felt that all this sum should not be expended on things useless to Mr. Griffiths, and, after ascertaining what would be acceptable to him, a bookcase suitably inscribed, containing a set of the *International Library of Famous Literature* (20 volumes), in three-quarter levant, was obtained; secondly, a silver bowl, with an inscription and a monogram; thirdly, an illuminated address, in a handsome Florentine frame, containing the names of the donors; also a book-plate, designed by Mr. W. V. Collette; and lastly, a cheque for the balance of the amount. The Committee have carried out these proposals, and the result is now before the meeting.[1] At the top of the address is an ornamental device founded on the seal of the Bristol Medical School. As Mr. Griffiths was an old Bristol student, and as this Society always meets in the School, the device seemed a happy one. The seal consists of the City arms, supported by Æsculapius on the one side and by Hygeia on the other, and was originally designed by Dr. George Downing Fripp. The motto of the scroll has been altered for that of the *Journal*—

"Scire est nescire, nisi id me
Scire alius sciret."

This came from Lucilius, who wrote more than a century before Christ, and Dr. Smith believed that Mr. Griffiths had something to do with its selection. The illumination of the address has been done in a way which had met with the Committee's entire satisfaction, and it was certainly enhanced by the Florentine frame by Messrs. Frost and Reed. Before he sat down, Dr. Smith said he would like, as Editor of the *Journal*, to say a word. No member of the Society was in a better position than himself to realise what Mr. Griffiths had done for them, and no words could express quite adequately their appreciation of his work.

Dr. J. E. Shaw said that he would not like the occasion to pass by without saying a word, as Chairman of the Committee of the Bristol Medical Library. Nothing could surpass the excellent and noble work which Mr. Griffiths had carried on for the profession for so many years. Dr. Shaw had been associated with Mr. Griffiths in a literary way ever since he came to Clifton. In more recent years Mr. Griffiths had bestowed his high intellect on the beautiful Library in which they now were. Their esteemed friend had worked for many years in an unselfish spirit, not to advance his own interests, but to advance the commonweal of the profession in the neighbourhood.

Dr. J. G. Swayne, the *doyen* of the local profession, bore his testimony to the value of Mr. Griffiths's services.

1 A print of the illuminated address, executed by Mr. William Bennett, forms our frontispiece, and the lettering is sufficiently clear to be easily read: the original, in colours, being a handsome work of art.

Mr. Nelson C. Dobson said he was very glad to avail himself of the opportunity of saying a few words on this interesting occasion. It was an unique occasion in the history of the Society. He had been a member of the Committee from its formation, and when he recalled their modest aspirations, he might say that their success had far exceeded anything they had in contemplation. This success was due to no particular member, but if he were asked to name one member who had contributed to that success more than any other, he would name Mr. Griffiths. The members of the Society have evidence of this in the magnificent Library around them, due to his energy and persistent industry. His work in connection with the *Journal* he (Mr. Dobson) knew had been a labour of love. No doubt, had Mr. Griffiths devoted his great talents and his great industry to other channels he would have achieved a still higher professional eminence, and have reaped a considerable harvest of pecuniary gain. His reward he finds in the satisfaction of work well done. He thought Mr. Griffiths exemplified in his own person the words of his favourite author, in which Orlando says:

> "How well in thee appears
> The constant service of the antique world,
> When service sweat for duty, not for meed!"

Dr. C. Steele remarked on the comprehensive work of Mr. Griffiths, in his capacity as Librarian, and on the great interest he showed in the members individually.

Mr. F. R. Cross said he could not too strongly endorse what had already been said. The profession in the West of England was indebted to Mr. Griffiths most particularly for this Library, which is largely due to his work and knowledge of the subject and the time he has given to it. Mr. Cross had long felt that if there was any member who deserved well of his professional brethren, Mr. Griffiths was that man.

The President stated that Mr. Griffiths had laboured for seventeen years for the *Journal*. During half that time, he had had the pleasure of serving on the Committee with him. Few had any idea of the amount of time which Mr. Griffiths had given to the *Journal*. His industry and devoted labours had contributed largely to the success it had attained. Nothing seemed too small for his research, and this went on from year to year, but there came a time when he found he must give it up. The Committee thought something should be done to mark the Society's appreciation of his services. The subject of a testimonial was brought forward, with the result laid before the meeting. The President then made the presentation.

Mr. L. M. Griffiths, on rising to acknowledge the testimonial, met with a very hearty reception. He said: Mr. President and Gentlemen,—During the last few months many an Englishman has found himself in a tight place owing to the malice of his enemies. To-night you have the spectacle of another Englishman in a tight corner from a totally different cause—not the hatred of his foes, but the kindness of his friends.

It is true that I cannot plead, like some of my countrymen who have been in difficult positions lately, that I have been taken by surprise, for apart from the very explicit statement in the circular convening this meeting, those who have been principally concerned in this to me very gratifying presentation, knowing that I am a person of feeble lips and stammering tongue, kindly gave me early information of the project. But I am not sure that in this instance being forewarned was being forearmed, for I feel nearly overwhelmed by the all too flattering observations which have fallen from those who have spoken to-night, together with the cordial way in which those observations have been received, and I have no words with which to adequately thank you and the other donors for these very handsome and useful presents.

This is an occasion when it is appropriate to briefly review the history of the *Journal* with which I was so intimately connected for seventeen years. The Society which is its sponsor, and through the efforts of whose members the *Journal* became a possibility, was founded more than a quarter of a century ago, largely by the untiring energy of Dr. Shingleton Smith, who became its first honorary secretary. He, Mr. Dobson, and I are the only members who have been on the Committee from the beginning.

Four years passed, and in 1878 it was decided that some of the good work which the Society had done should find a permanent record in a volume of *Transactions.* Accordingly a volume was issued which, considering the quality of the matter it contained, was published at the exceedingly modest price of five shillings.

Another four years passed, and some members of the Committee thought the time had arrived for the publication of a second volume of *Transactions* covering that period. Feeling that such a retrospect was at its best by no means satisfactory, and knowing well the ability of my professional brethren in the district, I had the temerity to propose in Committee that a publication with a more frequent periodicity, and having the nature of a journal or magazine, should be attempted. This proposition received the support only of Greig Smith and myself. But when the question introduced by Dr. Beddoe was brought before a general meeting of the Society it was determined *nemine contradicente* that the effort should be made, and in 1883 the first number of the *Journal* was issued.

It was fortunate in its beginning. In the person of Greig Smith it had an Editor who *con amore* placed at its disposal his commanding and masterly genius, and his professional reputation gave it an impetus which was invaluable. It had also in Mr. Arrowsmith a publisher who took far more than a commercial interest in its welfare, and his local patriotism, so well known in many directions, resolved that it should take a form worthy of the profession which it represented. In this he was ably supported by his staff, many of whom have worked at it during the whole of its existence, and with whom it has been a pleasure to me to be associated in the position with which you honoured me.

The second number appeared after an interval of six months, but after the first year it became and has remained a quarterly publication. Before long it ought to become a monthly one, for there is enough local talent and energy to accomplish this.

Its professional success has been great. This is partly testified by the fact that its contributors often find abstracts of their work chronicled in various countries of the world, and partly by its large exchange-list, for, with the exception of some few journals of a special nature which exchange only with their own kind, this includes most of the best medical periodical literature extant. An examination of the exchange-list and of the list of subscribers shows that the *Journal* now travels over the whole globe.

It was a financial success from the first, and it was mainly owing to its accumulated funds that we were able to start a Library in 1891. The part it has played and is playing in the formation of this Library is a considerable one. Publishers and authors have shown a strong desire to get its opinion of their books, and a special debt of gratitude is owing to all our reviewers for so generously allowing the volumes which they review to come to the Library for general use.

My journal-career has shown that in the work of such a publication there is room for the man of small parts. A successful medical journal must have at the head of its affairs an editor with a well-balanced mind, and among its staff and its contributors those who have clinical and literary accomplishments; but, in addition, it must have someone who will look after its business affairs, see that its matter is methodically arranged and in something like right proportions, and that it goes out with every "i" dotted, and with every "f" unbroken. It is because the *Journal* now has all these concerned in its direction that its success is assured. In some of the departments I have, of course, gained considerable experience, and if that experience is of service to anyone, I shall only be too glad to place it at his disposal. But the worth of experience is often over-rated,—especially by the possessor of it. It gets too much enamoured of itself, and it frequently gets too much deference paid to it. If it is only the experience of a mere plodder, and is not supplemented by an informing and advancing intelligence, it is tempted to disregard, or at all events not set sufficient store by, that cleverness which is—in the words an Australian quack doctor employed to describe his diagnosis—both intuitive and instantaneous.

Although my official connection with the *Journal* ceases, I am glad to say that I shall still be somewhat in touch with it, for the Committee have honoured me by asking me to continue to supply what I am given to understand are not its least acceptable pages; but I part from the greater work with many regrets, for though it occupied more time than perhaps I was justified in giving to it, it was a labour in which I delighted. I have felt all along that the professional life of a district is the richer for a *Journal* which worthily gives expression to that life. To that end I laboured, and it is peculiarly gratifying to me to receive the commendation of those who have been close observers of my work, and who have been brought into touch with it almost day by day.

I regret that my vocabulary is not equal to the task of recording my gratitude for the handsome way in which by word and deed you have acknowledged my *Journal* work, but in the absence of eloquent words in which justice should be done to an occasion like this, I ask you to accept my heartfelt thanks for all the sympathy and kindness you have shown me.

## Figures and Tables

**Figure f1:**